# A Christmas Carol in a Minor Key

**Published:** 1888-12-29

**Authors:** 


### A Christmas Carol in a Minor Key.

"Hark! the herald angels sing
Glory to the new born King ——"

"Bother those children!" I exclaimed impatiently, throwing down my pen. I was just finishing my article on "Intelligent Selfishness," for the January number of the "Individualist," and the children outside my window would insist on interrupting me by chanting snatches of Christmas carols all day long.

"They don't mean no harm," said my housekeeper when she brought in my coffee, and found me walking up and down the room in a state of intense nervous irritation. "It's natural people should be happy when Christmas is at hand, and when children's happy they 'most always sing."

Perhaps they meant no harm, but they interrupted my work as much as if they did; and the article which I had on hand was a very important one, and required the utmost concentration of thought. I had looked at the subject in all its bearings, and though I was perfectly convinced of the accuracy and morality of my views, I knew that it was uncommonly difficult to state them in such a fashion as to impress their truth on the somewhat emotional mind of average humanity.

The Gospel of Selfishness.

My theory, in brief, was this: If everyone would only look after himself there would be no need for other people to look after him. If every man did his duty to himself we should have no paupers; if every man worked as energetically as he might, there would be a marked increase of national prosperity. The consistent practice of intelligent selfishness would do away with the necessity for hospitals, workhouses, and refuges of all sorts; and, according to my views, the giving and receiving of charity were equally degrading to the character, tending, in the one case, to the development of laziness, and, in the other, to the deification of self-conceit. The charities of which people boasted were no evidences of advancing enlightenment, but the survivals of a past and imperfect civilisation. Under the *régime* of intelligent selfishness the struggle for existence would probably be more severe, and the weakest would be driven to the wall rather more promptly and forcibly than they are now; but, after all, that was the best place for them. At present, they only stood in the way of their betters; and the improvement of the human race could only be attained by the speedy and total elimination of the unfit.

However sound these views may be, it is needless to say that they are exceedingly unpopular—at least, when stated in so many words—and till the "Individualist," was started, with an editor after my own heart, I had not found an opportunity to express them in such a manner as to gain the suffrages of the public. Therefore it was the more annoying that now, when my chance had come, and I was sailing through my subject on the full tide of thought, I should be distracted by those wretched little urchins chanting their old-world songs about herald angels and watching shepherds. If the law had permitted it I should have thought it an act of intelligent selfishness to emulate the doughty deeds of King Herod.

The Thread is Broken.

Failing that extreme measure, what was I to do? The thread of my thought was broken, and as I did not wish my peroration to be lame, it would, I felt, be wiser for me to go elsewhere till these carol-singing children had gone to bed. I put on my hat, and wandered aimlessly out into the streets, which everywhere displayed the conventional signs of Christmastide. Not that I cared for Christmas! My pudding-eating days were long time past, and the thought of goose infected my imagination with dyspepsia. It seemed centuries since I had meddled with snapdragon. I remember the last time, in the old home that has passed to strangers now, when we protested that Cousin Will did not play fair because he was using a spoon instead of his fingers to snatch the raisins from the flaming dish. Poor Will! he tried to get the plums of life by illegitimate means, in more serious matters, and—well, we were glad for the sake of the family credit, to pay his debts and pack him off to California, there to sink or swim. Poor old boy! he had his merits, too: was always good-tempered and cheerful; the little ones adored him. But why should I pity him? He brought his fate on himself, and have I not said that the weakest must go to the wall—the weakest in moral, as well as in physical and intellectual force? Yet, "for old sake's sake," I would like to know what Will is doing this Christmas Eve.

A Chance Step.

This Christmas Eve! Can I not forget the traditional festival? Apparently not, while I wander thus alone. What a crowd of people there are going up the stairs to this hall! I know the place—the chosen spot for public meetings and public dinners. Perhaps there's something going on now. If I can obtain admittance it may amuse me for an hour.

Getting in was easy enough, but what had I come upon? A bazaar, a toy-shop, a doll-show? In the name of all that's intellectual, what was I doing there? Well, chance brought me. But there's no such thing as chance. I am at one with the theologians on that point, though my explanation of the matter is different. They say that what we call chance is the action of providence; I say that it is the operation of some yet uninvestigated law of the universe. And I must admit that this yet uninvestigated law of the universe had brought me on a pretty sight.

Among the Toys.

I am still, though I did not think it, woman and child enough to like a doll, and there was plenty of variety here to choose from. Most fashionable fine-lady dolls in scores and hundreds, dressed to go out, to go to a ball, to go to Court—fan, feathers, veil, and all. Patient and loving fingers must have worked at those lilliputian garments, especially those where the wearers were labelled, "Clothes to take off and on." The grammar of the phrase may not be perfect, but, oh, the fascination of the idea! The endless delight of putting your doll to bed a dozen times a-day, and waking her up again before she has time, even if she had the power, to close those bold blue eyes of hers. A child is never quite desolate who has a doll. It is the companion of lonely hours, the heroine of day-dreams. What marvellous adventures my dolls had! What hairbreadth escapes (sometimes not without injury to the wax of their noses and the sawdust of their chests) from giants, dragons, and pagan kings. It is rather pitiful, after all, to think that in the millennium of materialism, when children will be brought up on a diet of facts as hard and wholesome as dried peas, our girls will no longer be permitted to weave romances round their dolls.

A Plea for the Dolls.

Nay, we'll squeeze the dolls in yet, if they can be made as instructive as the puppets here. For in this room, with the winter sunlight falling through its great windows on the pyramids and thickly-planted banks of dainty toys, one might study history, geography, and literature, all exemplified by dolls. Mary of Scotland stands amicably by the side of her life-long foe Elizabeth, while near them, regardless of the centuries, are subtle Cleopatra, hapless Margaret of Anjou, and still more hapless Marie Antoinette. Then here is a wide plateau of peasant girls—Spanish, Norwegian, Swiss, Italian, with Eastern slaves and Scottish fishwives—a perfect gallery of national costumes. And near by are Shakespeare heroines

—"pretty maids all in a row"—Portia wearing her doctor's gown a shade too coquettishly; Rosalind marked by an air of slight discomfort in her woodland garb, that suggests the idea that she is at that moment exclaiming, "What shall I do with my doublet and hose?" and Juliet, kept from falling into Romeo's arms, not by the balcony's height, not by the harsh command of her stern sire, but by—a piece of string!

Yes, they were all very quaint and pretty, but on the whole I liked this big doll, dressed as a "long-clothes baby," best. So apparently did a little girl, who with her mother was looking at the toys, her mouth and eyes various-sized "oh's!" of admiration. She stopped before this masterpiece of dainty work, and gazed at it with approving eyes.

### Best of All.

"I like that best of all," she said, with a fine air of decision. "I hope they'll give that to a little girl who is very, very ill."

"Why, dear?" asked her mother.

The little maid looked slightly embarrassed.

"Oh, when one's ill one wants a big dolly, somehow," she said, vaguely.

"But I don't understand why," persisted the mother.

The child hesitated for a moment, and then said, blushing slightly, with a quaint, pathetic shame—

"Well, I'll tell you, mamma. You remember when I was ill, last year—when my throat hurt so much—I always wanted my biggest dolly in bed with me."

"Yes, darling, I remember."

"Sometimes I couldn't help crying, the pain was so bad, but I didn't want you to know, because you looked so troubled when you heard me; so I used to hide my face in dolly's frock and cry quietly, and you often thought I was asleep."

The mother stooped, and, under pretext of pushing back a stray golden tress, kissed the earnest little face.

"I didn't know that," she said. "You were a brave little girl, but, all the same, you need never mind mamma knowing when you are in pain. I want to help you to bear your troubles."

I strolled on, somewhat moved from my philosophic calm by the conversation I had overheard. "There's some excuse for toys," I said to myself, "if they help, in ever so slight a degree, to give courage and cheerfulness to sick children."

### Sick Children.

After all, we must do something for them, let the political economists say what they will. They suffer, not through their own fault, but as a result of the ignorance, the selfishness, or the neglect of others. From the point of view of physiology, one may say of some of them, without a thought of harshness, that it would have been better if they had never been born; but having been born, and having made a struggle to survive against very adverse circumstances of home and constitution, it seems only fair to give them a helping hand.

I was by this time moved to some curiosity about the *raison d'être* of this great collection of toys, and on making some inquiries, I learned that these were the *Truth* toys. The proprietors of the journal *Truth* offered prizes to a considerable amount for the best toys of various kinds, and distributed the playthings thus received among the children in the hospitals and workhouses of London. Some of the dolls I had been looking at were sent for this competition, but the greater number of them, with the cricket-bats, tea sets, miniature shops, kitchens, and locomotives, and miscellaneous toys of all sorts were the freewill offerings of kindly souls. Others sent contributions in money, which enabled those who got up the distribution to buy rocking-horses and other expensive articles for presentation to hospital wards as permanent playthings for the quickly succeeding generations of occupants—playthings kept on communist principles—nobody's property, because they are everybody's.

### Fact versus Fancy.

I was interested in the sight I had thus by accident come upon, and began to speculate upon the fate of these toys, and the little sufferers who were to receive them. It would be at least a new experience, interesting from a scientific point of view, to see how far the purely imaginative pleasure of possessing a plaything would overcome the stern, material facts of weakness and pain. Can mind triumph over matter? A little thing may answer the question as well as a great one. Gravitation is shown in the falling apple as clearly as in the avalanche; and if a sick child can for a moment be uplifted from the dreary consciousness of pain by the lights and glitter of a Christmas-tree, it is easier to realise the hero's devotion, the marty's rapture, which annihilates present pain by the sure hope of future joy. That is a wide statement to evolve from a plaything put into a thin and feverish little hand, but in a world so full of analogies as ours, there is no great fact of humanity that is not implied and foreshadowed by a hundred little facts, each showing the instincts of the body and mind of man in miniature, and he is no true philosopher who ventures to ignore them.

### Why One Avoids the Hospitals.

I had never been in a hospital in my life. Following out the doctrine on which I had moulded my life, I had reasoned that to see suffering which I could not help was only causing myself a totally unnecessary discomfort. Now, I felt a strange curiosity to witness this unpleasing sight I had so long evaded. I should not enjoy it. No, I was sure of that; but, somehow, I wanted to see it. Here's another eccentricity in human nature—to desire that which will grieve one's heart. I began to find a new interest in my own mental processes. I thought I had analysed all my emotional possibilities long ago, and here were new developments. I made inquiries, and set to work to trace some of the *Truth* toys to their destination.

### At a Children's Hospital.

With very little trouble, I got permission to be present at the Christmas festival at a large Hospital for Sick Children. Here it had formerly been the custom to have a great Christmas tree in the hall of the hospital, but this arrangement not only caused unnecessary danger to the children from fire and draughts, but prevented those who were confined to bed from sharing in the fun at all. So, as other changes had taken place during the year, the secretary had taken the opportunity of making a change in the management of the Christmas festival. A Christmas tree was set up in each ward, and every inmate felt a personal interest in the matter. The gifts from the tree, as well as the more solid presents that lay around, were distributed with as much justice as was possible. Comparisons were many, but on the whole satisfaction prevailed. The experienced ones, who had been in hospital before, compared this festival with others, both here and elsewhere. One must have something to boast of; even a grievance is better than nothing; and in a hospital, especially among children, they are much inclined to boast of their ailments. Thence come envyings and strife; pneumonia looks down on bronchitis, and he who owns a broken leg despises both. These are subjects of conversation for every day; but at this time I heard animated discussion of the respective charms of tea-sets and sheep-farms, together with calculations as to whether it was better to be in a children's hospital, or in the children's ward of a general one.

### The Crown of All.

Opinions differed, and each little patient with any pretension to knowledge vaunted the experience he or she had known. But a demure damsel of some nine years settled the question.

"I was here before," she said. "It was in the summer, and one Sunday the Princess of Wales came round and gave us all flowers. Mine were tied with a ribbon—a ribbon she had worn, mind."

She looked round on her companions with the calm self-satisfaction with which a lady who has just been presented at Court flaunts her train and plumes before her less honoured or aspiring friends. There was silence, no one could recall an incident to compare with this. "I've got it now," added the favoured one; and after this there could be no further question of the superiority of this hospital to all others under the sun.

### In the East.

From Great Ormond Street I went to a large hospital in a poor locality, moved chiefly by a desire to see if the little East-enders fared as well as their comrades of the City. Even genial Father Christmas, I thought, might excuse himself from visiting Whitechapel, when his advent was awaited so anxiously in more inviting quarters. When, however, I got within the big grey building, I saw unmistakable signs of festivity, which grew more marked as I approached the children's ward, which bears the name of that good mother and lover of children, Queen Victoria. I found that Santa Claus visits the ward in orthodox style. Whether he comes down the chimney with his bag of toys I am unable to say, and I found that a certain heresy concerning the manner of his appearing was prevalent among the occupants. The general belief seemed to be that he was secreted in "Sister Queen's" room at the end of the ward, whence he came out in the small hours to fill the tiny stockings that gaped with curiosity and expectation before him. Into each were put sweets and such minor toys as they would hold, while larger presents were distributed by the nurses till the whole ward was vocal with the lowing of cows, the barking of dogs, the mewing of cats, and the stiff "mamma—papa" of mechanical humanity—all bearing a family likeness in point of intonation. Meanwhile puzzle maps were dissected, scrap-books opened and soiled, and soldiers set forth in marching array, all with the marvellous promptitude of youth.

### Music and Dancing.

But the height of joy was reached when an organ, or, rather, a street piano, was introduced into the ward. Looked upon simply as a street piano, I considered it incomplete; in my own youth I always rather grudged a penny to an organ that had not a monkey to take the coin. In a general way the monkey may be regarded as an essential part of the instrument; but here it could well be dispensed with, for at the sound of the "Boulanger March," a score of quaint little figures, dressed in all manner of invalid attire, "took the floor" and danced with a hearty enjoyment and an originality of step I had never seen equalled.

"Why aren't you dancing?" I asked one bright eyed little girl, whose hands were beating a tattoo in time to the music on the counterpane of her cot.

"I can't, 'cause of my china leg," she explained; but the explanation was a thing of mystery to me, till a nurse informed me that the "china leg" was a euphemism for a leg wrapped up in a plaster-of-paris bandage. She had to remain in bed, and so had some others who had limbs in splints, but they wriggled and writhed and shrieked with a delight almost as keen as that of the dancers.

### Fun for All.

When the children's festivities were over I made my way into the adult wards, where different kinds of entertainments were going on. In one a conjurer was amazing the patients, in another a magic-lantern entertainment was going on. In a third was a lady whose face and name were familiar to me as they would be to most theatre-goers. I had often admired her talent, but it seemed to me that she had never laid her finger on human emotion with so sure a touch as now, when she recited one of Sims's ballads. "Dagonet" may not be exactly a poet of the highest rank, his style would hardly suit any epic but that of the slums, but he has one merit that is often lacking in more pretentious bards—he is eminently suited for recitation. With a popular audience he is sure to take, and, on the whole, when you are addressing a popular audience that is what you want. I was not sorry that "for this occasion only" the clever reciter condescended to "Billy's Rose." It was a women's ward, and the story seemed to touch some of the listeners almost too keenly, but when afterwards they heard Mrs. Caudle's lecture on the crime of lending an umbrella, delivered with a point and vigour which Mrs. Caudle herself might have envied, they displayed an exquisite appreciation of every point. If I thought I detected in the laughter a touch of self-consciousness, as if, save for the humour of it, that style of addressing a husband was not wholly unknown to them, it is likely enough that the suspicion was wholly unfounded.

### A Change of Temper.

By this time I had become interested in my self-imposed task. Hitherto I had avoided hospitals and all similar institutions. I did not approve of them, because I held that they tended to perpetuate weakness that had better perish off the face of the earth. But having made the plunge my ideas had changed considerably. Perhaps my principles had fallen into the background, for I forgot everything but the interest, not wholly painful, I felt at the sight of these long wards, the wonderful cheerfulness shown by the patients—made less wonderful, however, by the unwearying care and even courtesy of the nurses, and the trouble that had been taken to give a bright and home-like air to all the surroundings. Everywhere I went, among enumerable differences of detail, I found the same marked characteristics—a determined joyousness that, for the time at least, drowned care, aided by every mark of rejoicing that could remind those who saw it of the season of good will to men. Many willing fingers must have suffered the mild martyrdom of holly prickles in decorating these walls, and it is likely that many feet were weary before the last touches were put to the Christmas tree; while the difficulty of adapting Christmas fare to invalid digestions must have taxed the brains and tempers of cooks and matrons generally.

### Here and There and Everywhere.

Of course, every institution had its own special "attraction," to use the language of theatrical advertisements. At one institution a popular comedian spared an hour before his evening engagement began to delight a gathering of semi-convalescent patients with songs and readings; at an ophthalmic hospital, where most of the audience had at least one eye bandaged, the entertainment was a concert of really good music, listened to with an attention and enjoyment which perhaps the performers recalled with longing in more conventional concert-rooms. At another concert the committee scored" by having some nurses among the vocalists. For the average patient is more loyal than critical, and would rather hear his nurse sing a simple ballad, than Madame Patti trill the most elaborate aria in her répertoire. They knew that at the large institution where a choir of probationers went round the wards singing carols. At one well-endowed hospital there were elaborate *tableaux vivants*, beyond the power of those which depend on voluntary contributions. The pictures were shown within a big gold frame; and as those who took part in them were for the most part artists' models, they sustained their difficult poses with a statuesque stillness which, to confess the truth, gave common humanity the fidgets to witness.

### What Would the Subscribers Say?

It is needless to narrate all my wanderings. Everywhere I went I found the same brightness and kindliness that took the sharpness off pain and deadened trouble for the moment.

"But," I said to one official, "I don't know that the

Public subscribes money to be spent for the amusement of the patients in our hospitals. It is meant for their cure."

It was at a large and important special hospital that I made the remark, and the secretary answered promptly.

The Plea for Amusement.

"It takes more than medicine to make a cure; if you are in any degree a student of human nature you must know that, and all who know anything of our work will bear witness to the necessity of rousing the patients from the languor that is apt to be engendered by the inevitable routine of hospital life. At Christmas time especially, when the most fortunate of us are apt to be haunted by sad thoughts as well as joyous ones, patients need to be kept from brooding. Some of them, no doubt, had festivals at home a year ago, and their minds float back to these, and fret over their absence now. And, on the other hand, if they never had any such enjoyments, it does them good to learn that life is not all toil and weariness. Believe me, if we took off the Christmas treats, we should in many cases have to keep our patients longer in hospital, which would be a poor economy. In spite of this materialist age of ours, we who are connected with hospitals are forced to recognise the influence of the mind on the body. Believe me, the most serious results flow from causes that are intangible to our five senses."

The Philosophy of Christmas.

"Ah! you philosophise about your Christmas tree," I said. "Philosophy covers a multitude of—shall I say caprices? You can defend almost anything on philosophical grounds."

"I'm not sure of that," was the reply, "and I think I was talking physiology as much as philosophy. I was, if you analyse the matter, pleading for wholesome exercise of the brain, full and regular motion of the heart, and good action of the liver. But if I should philosophise, as you call it, my plea would be the same. I will, if you like, throw over the Christian origin of our winter festival, and take it as the heathen Yule, celebrating the beginning of lengthening days—the first dim promise of the spring What does it matter? Whatever you take as its history, you are confronted with the fact that human nature, in its most primitive condition, felt the need of a season of rejoicing, of an occasional throwing aside of the burden of care, of a time of peace and goodwill. The feeling is fundamental, instinctive; a demand that must be gratified. You remember what the rebellious Romans demanded of their Emperor—*panem et circenses;* bread and the circus. We may be less bloodthirsty in our tastes now, but we are still so far the same that food and amusement are alike necessary to our health. You won't deny that, surely—you who belong to the sex which is so often driven into melancholia by inaction.

I protested against my womanhood being brought in evidence against me. Yet I recalled the languor of a long convalescence, when it really seemed a matter of importance that I should have flowers in my room, and when the expectation of a friend coming to play *bézique* with me caused me the greatest excitement. (I hate *bézique* when I am in health, I may say.) And I admitted to myself that, though under ordinary conditions our work may supply us with all the interest we need, in time of illness, when even the stronger sex are "unmanned," a little amusement is a very good thing.

Where the Money Comes From.

"Still what do your subscribers say?" I asked.

"They don't say anything, for as a matter of fact their entertainments don't as a rule come out of the ordinary funds of the hospitals. we have always a few friends who remember the old couplet about Christmas coming but once a-year, and help to provide the good cheer. As for the decoration of the wards, the nurses look after that, and spend a good deal of time and often a little money in making them pretty."

"The nurses! I didn't know that they were well enough paid to have money to spend in that way."

"They are not, but they do it. You won't make much of a nurse of a woman who has not a housewife's pride in making her ward look bright and homelike, and as pretty as she can, as well as a doctor's pride in curing her cases. So you'll always find our good nurses ready to give a little out of their scanty salary to make their decorations successful."

Accomplished Nurses.

"Then the painted walls I have seen in one or two places, and the decorated door-panels—"

"Are all voluntary work. If a probationer comes who can handle a brush, you soon find her using all her spare time in decorating her ward; if she sings you will have a constant source of amusement for the patients. Other things being equal, it pays a hospital to have accomplished women for its nurses."

"Other things being equal," I echoed.

"Of course, what you primarily want in a nurse is a woman who can look after her patients, and obey the doctor's instructions intelligently. That is what the hospital pays for, but it isn't proud, and is very glad to take anything extra, such as charm of manner and general culture, as a gift."

The Fascination of Hospitals.

"But you'll make your hospitals too attractive. I don't quite approve of that you know."

"There is a danger, I admit; and one of the permanent troubles of hospital officials is the patient who can afford to pay and tries to get in for nothing. It is a wrong to our really poor patients, a wrong to our subscribers, and a wrong to our medical practitioners. But we are honestly trying to cope with it; some hospitals employ a man to investigate the claims of applicants for hospital treatment; others demand of the applicants themselves the proof of their inability to pay a doctor. It is a difficult business, but if we keep exerting ourselves, and at the same time try, both inside and outside the hospital walls, to rouse in people that sturdy, if rather sullen, self-respect which would make them scorn to take as a gift what they could afford to pay for, I have no doubt we'll get to a more equitable state of affairs in time. As for making them too attractive to those who are really under treatment, I don't think it is possible to do that. We have got to cure these people by any means in our power, and, as I said before, the sooner we do it the cheaper it is done. If you look at the diet sheets I don't think you will consider the meals luxurious. They are certainly not better than you are accustomed to; if they are better than the patients have at home—when they have a home and regular meals at all—you must remember that in many cases starvation is a great part of the disease, and an ample supply of wholesome food an essential part of the cure."

Are Hospitals Extravagant?

"Then all that we hear about hospital extravagance is untrue?"

"I don't say that, but it is grossly exaggerated. Not wilfully, I admit; but people who do not understand how many things are required to make a really efficient hospital, catch hold of an apparently large rate per occupied bed, and at once raise a cry of extravagance, if not of misappropriation. I assure you that we all wear out our lives in trying to effect economies in one department or another. A friend of mine, who is always mourning the great expenditure of his hospital, said he had examined the cost of housekeeping in every detail, and he couldn't get the cost of food below 8¾d. a-day for each individual. Now do you, as a woman, consider that extravagant?"

"I wish he would tell me how he gets it down to that," I answered. "I would publish the details for the benefit of the poor and struggling middle-class. But is there not extravagance in other departments?"

"To quote again from my friend's experience—he says that the business expenses are five per cent. of the income; in plain figures, that the salaries of secretary and clerks amounts to £500 a-year, and the income—requisite income to support the hospital—is £10,000. I think that is not excessive, if you compare it with the working expenses of other institutions. There is also a collector, who receives a small commission on the amount he brings in, but I need not tell you that if people would send their subscriptions to the hospital without waiting to be called upon, the collector's occupation would be gone. Mind, I do not say that there is not blundering and extravagance in hospital expenditure. Matrons, secretaries, and committees are apt to get into a routine, and give contracts to the people who have had them before, without inquiring as closely as they might if they are getting as much as they can for their money. But they are, speaking generally, anxious to do the best they can with the funds confided to them, and what they need is the opportunity of meeting to discuss and compare their expenditure."

"And quarrel over it, perhaps."

"A quarrel between honest men doesn't do so much harm as you might expect; they usually find a common ground of agreement after all. We have our vanities, of course. We all think that we alone have attained the ideal combination of comfort and economy, and don't like having the notion knocked out of us; but on the whole we would risk a hard blow, if we learned a wholesome truth by it."

### The Result of Rash Accusations.

"Still, these continual accusations force one to entertain a certain distrust of hospitals," I said.

"It is always easier to entertain a lazy distrust than to inquire into the justice of the accusation on which you found it."

"You are severe, but there is some truth in what you say. But if our hospitals, however well administered, tend to pauperise those who profit by them, I can't help disapproving of their existence."

"Hadn't you better extend your disapproval a little farther?" said my companion,—"extend it to the pain, disease, and misery that call our hospitals into being? They are mostly caused by human ignorance and sin, and so far I disapprove of them too. All the efforts of our generation are directed to the annihilation of these two factors of disease; and the hospitals, through their medical schools, do their share of the work. But neither ignorance nor sin is dead yet, and we have to deal with their results. What we try to do is to modify these result as much as possible. Do you disapprove of that?"

"No; but yet it would be better if pain and weakness perished off the face of the earth."

### The Meaning of Pain.

"Are you sure? When I look round our wards I sometimes feel very bitter against the things that send our patients to us—the fiends of drunkenness, vice, unhealthy employments, insanitary houses, and all the other things that strike men down with illness. But then there recur to my mind these lines of Longfellow's:—

'It is Lucifer, the son of mystery;
But since God suffers him to be,
He too is God's minister,
And labours for some good
By us not understood.'

"An old-world doctrine," I exclaimed sadly, "that will scarcely satisfy a modern critic."

"Because the modern critics think they understand everything, I suppose. And yet the enormous extent of modern discovery should teach them, by their own scientific methods, to infer an immense amount more to be discovered. There is a friend of mine who is such a thorough-going evolutionist that he believes that in a few more generations the human race will have advanced so much in purity and strength that it will indeed see God, as Enoch did of old. That seems fantastic to you, no doubt, but it is the logical outcome of that gospel of advancing humanity which so many preach."

I shook my head. "It will never come to that."

"No; not if the materialists get their own way. It is they who form the inquisition of these latter days, and we poor Galileos, who believe that the world moves in its orbit according to the will of a Higher Power, are forced to say our *è pur si muove* very low indeed."

"We are straying from the point," I said.

### Who are the Fittest?

"Which was the preservation in life of the sickly and infirm by means of hospitals, I think. Of course, you believe in the survival of the fittest—that's what you are going on, no doubt. But surely this intellectual age, which I will assume that you represent, doesn't believe that physical fitness is the one thing needful. That it is needful I admit; we feel that so deeply in hospitals that our main effort is to replace it where it is lost. But it is not everything, and we owe too much to those who have suffered from physical infirmity—shall I venture to say from the Apostle Paul, with his "Thorn in the Flesh" to "The Lame Boy," Lord Byron—to return to the prize-fighter ideal of humanity. In talking of the survival of the fit, it is well to remember that those who are comparatively unfit for present conditions may be ideally fit for the higher conditions of the future. We cannot tell; working in the dark as we do, it is wiser to give every creature its chance of prolonged life, which may mean prolonged usefulness."

"We are getting into deep water," I said. "But you certainly justify your cause."

"Do you not justify it too? What have you seen in our hospitals?"

### What Hospitals Teach.

"I have seen," I answered slowly, "the pain and disease which are inseparable from the struggle and stress of our present conditions of existence tended and relieved with skill and care; I have seen more cheerfulness in the wards than I have in the homes of the poor; I have seen rough natures softened and refined by illness, and fine natures strengthened and ennobled by tending others; I have seen the comforts of life, which we would fain bestow on all, extended to those who would suffer most from the lack of them; I have seen—yes, I have seen the ideal we seek—the Brotherhood of Man."

"And are these things worthless? You will hardly say that under our present conditions they are unnecessary; when they are, and there is no longer any use for hospitals, I—if I am alive then—will gladly see them turned into technical schools or anything else the wisdom of mankind may choose. Till then ——"

### The Practical Lesson.

"Till then," I said, "you may set me down among your annual subscribers."

"Ah!" exclaimed my companion, with an air of satisfaction, I have always said that women have very practical minds."

I laughed at the compliment, and took my way home. I had spent several days in my wanderings, and the year was drawing to a close. My curiosity had, I confess, made me neglect my ordinary work, and on the day after the conversation I have recorded, I received an anxious letter from the editor of the "Individualist," asking me to send at once my article on "Intelligent Selfishness." I answered thus:—

### Good-bye to Selfishness.

"My dear Sir,—The paper on 'Intelligent Selfishness' is unfinished; it will never be finished now. I have changed my opinions, and now believe selfishness to be the most stupid thing in the world, the heaviest drag on the wheel of progress. There is but one wisdom—that mutual love and help which draws humanity together, and leads them unconsciously to a higher life. By teaching them love, not by teaching them selfishness, can we help our brothers to bear their burdens and to live their lives for the good of all."

My letter posted, I sat down to think, and from the fading Christmas holly—by the way, isn't that name a contraction for "the holy thorn"?—my mind wandered on to the Easter lilies the spring would bring. I thought of that mystery of birth and death around which a world's faith has gathered, and I remembered St. Paul's words, "The Captain of our salvation was made perfect through suffering." I remembered, too, the sponge soaked in vinegar which was pressed to the agonised lips on the cross—the "criminals' drink," it is said, given to deaden the agonies of crucifixion—and I recognised in that supreme instance help, however small, given with pity and received with gratitude. Down through the ages there came to me the conviction that suffering was part of a Divine intention, and that the spirit of Him who went about healing the sick still walked the earth with those who in one way or another, strove to aid such of their brethren as have fallen by the way.

Poverty and pain are with us still. How long they will be in our midst we cannot say; but it requires no great wisdom, no miraculous prescience, to see in everything that strengthens, heals, and comforts humanity, a step towards "that far-off divine event to which the whole creation moves."

